# Capturing At-Home Health and Care Information for Children With Medical Complexity Using Voice Interactive Technologies: Multi-Stakeholder Viewpoint

**DOI:** 10.2196/14202

**Published:** 2020-02-13

**Authors:** Emre Sezgin, Garey Noritz, Alexander Elek, Kimberly Conkol, Steve Rust, Matthew Bailey, Robert Strouse, Aarti Chandawarkar, Victoria von Sadovszky, Simon Lin, Yungui Huang

**Affiliations:** 1 Research Information Solutions and Innovation The Abigail Wexner Research Institute Nationwide Children's Hospital Columbus, OH United States; 2 Department of Pediatrics Nationwide Children's Hospital Columbus, OH United States; 3 Family Advisory Council Nationwide Children's Hospital Columbus, OH United States; 4 Care Coordination and Utilization Management Nationwide Children's Hospital Columbus, OH United States; 5 Professional Development Nationwide Children's Hospital Columbus, OH United States; 6 Department of Biomedical Informatics The Ohio State University Columbus, OH United States

**Keywords:** care coordination, self-management, children with medical complexity, voice technology, voice assistant, digital health, conversational agents

## Abstract

Digital health tools and technologies are transforming health care and making significant impacts on how health and care information are collected, used, and shared to achieve best outcomes. As most of the efforts are still focused on clinical settings, the wealth of health information generated outside of clinical settings is not being fully tapped. This is especially true for children with medical complexity (CMC) and their families, as they frequently spend significant hours providing hands-on medical care within the home setting and coordinating activities among multiple providers and other caregivers. In this paper, a multidisciplinary team of stakeholders discusses the value of health information generated at home, how technology can enhance care coordination, and challenges of technology adoption from a patient-centered perspective. Voice interactive technology has been identified to have the potential to transform care coordination for CMC. This paper shares opinions on the promises, limitations, recommended approaches, and challenges of adopting voice technology in health care, especially for the targeted patient population of CMC.

## Introduction

Immense efforts have been placed on capturing health information electronically, thereby modernizing health communications. The majority of these efforts are provider driven and center around traditional clinical settings. However, a lot of health and care activities happen outside of clinical settings and are not systematically documented and integrated into the clinical systems. Such a practice limits the information captured per patient, which may lead to adverse effects in clinical decision making. This is especially concerning for children with special health care needs (CSHCN). CSHCN is defined by the federal Maternal and Child Health Bureau (MCHB) as children who have or are at an increased risk for chronic physical, developmental, behavioral, or emotional conditions and who also require health and related services of a type or amount beyond that required by children generally [1]. About 23% of US households have at least one CSHCN [2], and their needs include, but are not limited to, prescription drugs (86%); specialty care (47.5%); vision care (35.3%); mental health services (27.6%); occupational, physical, and speech therapy (26.6%); medical equipment (11.3%); hearing aids or care (5.2%); mobility aids (4.6%); and communication aids (2.5%) [2].

Children with medical complexity (CMC), a subset of CSHCN, have significant health issues that occur outside of the clinic, and they require complex home care provided by parents and other caregivers in addition to nurses [3]. CMC have medical fragility, medical technology dependence, functional impairment, and intensive care needs that are not easily met by existing care models [3]. Common characteristics of CMC are as follows: (1) they are one of the most frequently hospitalized populations; (2) follow-ups are more complex compared with regular patients, requiring multiple specialties; (3) they use multiple medications; and (4) they are more likely to have complications post discharge. These characteristics highlight the critical needs for effective care management outside of clinical settings, timely health information sharing, and sophisticated care coordination for CMC. The literature also supports the need to improve care coordination [4,5], which is the process of linking patients and caregivers to necessary services and resources in a coordinated effort for providing optimal health care [6].

A team (coauthors), consisting of caregivers of CMC; a clinician who specializes in treating CMC; a care coordinator who assists CMC and their families; a user experience designer; an application developer; and scientists and researchers who are experienced in clinical informatics, participatory design, and digital health, was formed at Nationwide Children’s Hospital. The team identified gaps in care coordination for CMC and their families, how various technologies can fill these gaps, and how they could be implemented and adopted, all from a patient-centered perspective. The team discussed the value of health information generated at home and the challenges and barriers associated with capturing that information. The team developed recommendations to improve not only record keeping of patient care at home but also communication among patients, caregivers, and care providers through technological solutions. The purpose of this paper was to present our opinions on employing emergent voice interactive technology to capture real-time health information and to enhance care coordination, the associated challenges in adopting this technology, and desired future development.

## Challenges in Care Coordination for Children With Medical Complexity

For CMC, the role of care coordination is highly valuable because the responsibilities (time spent, effort, and financial burden) are higher and navigating services is more difficult [7]. Most health-related events occur within a patient’s home. These include the occurrences of symptoms; medication administrations; home therapies; and, in the case of patients with technology dependence, the use of life-sustaining technologies such as ventilation, tube feeding, and intravenous medications. Highly relevant and valuable information generated in the home setting is not currently captured systematically in electronic medical records (EMRs), but it can be of great help in enabling effective care coordination and improving clinical decision making and treatment planning. Collecting relevant and complete health information at home is challenging. Some CMC might have physical impairments that prevent them from participating in clinical information gathering and decision making in a traditional way. Parents of CMC may be very busy with meeting routine and unscheduled on-demand care needs at home, making it difficult to consistently and accurately document or provide health updates. These situations suggest that there is a need to find a different approach that is easier for the patients and caregivers to provide relevant health information to the clinical team on a timely basis.

Helping CMC and families provide the right home-administered treatments at the right time, promptly documenting clinical events (medication, therapy, oxygen treatment, etc), recording symptoms as they happen, and reaching out for timely assistance are critical to promote self-management and coordinated care skills and for successful care coordination [5]. Within the scope of home care and use of technologies for care coordination, we identified 3 problematic care coordination gaps to be addressed: (1) untimely and incomplete capture of health events at home, (2) lack of home care coordination tools, (3) long term adoption problem for health care apps.

### Untimely and Incomplete Capture of Health Events at Home

It is commonly observed in clinical practice that patients and families do not have accurate recall of symptoms, clinical events, or usage of over-the-counter medications [8]. Patients and families also frequently delay reporting of time-sensitive health issues because of the burden of communication resulting from health care disparities [9]. Untimely and inaccurate communication with health care providers may result in misdiagnosis, mistreatment, extra visits, and extra cost [8]. To fill the gap, symptom tracking and monitoring apps have been developed over the years to help patients document health events outside of clinical environments [10-13].

...I couldn’t remember how many times I have given my daughter Albuterol treatments in the last two weeks…

Caregiver

However, many of these apps fail to promote timely documentation of health-related information because of cumbersome user interfaces, lack of functionality, or not providing evidence-based and personalized content. In many cases, the perceived value does not overcome the burden of using the apps [12,13]. Existing technology-based solutions are typically screen driven, requiring users to navigate through the hierarchy of symptoms with multiple clicks or touches to find the right place, then users may be presented with a prepopulated list of choices from which to select. They do not allow natural, unstructured recording of symptoms and clinical events. The time and effort needed to document creates a burden, which prohibits the adoption of these tools. It is particularly burdensome for CMC as they experience more symptoms and for longer periods. For instance, patients discharged from the neonatal intensive care unit might need to be closely monitored at home. Their conditions might change quickly, heralded only by fluctuations in heart rate or oxygen saturation, with illnesses ranging from a minor viral infection to a bowel obstruction. Caregivers might have their hands full when symptoms occur, making it difficult to record real time, especially if typing is required. Therefore, documentation activities are postponed and potentially forgotten while providing care, which can lead to adverse events and low or inaccurate recall of events when communicating with the clinical team. An integrated, easy-to-use, real-time, low-burden tool for health logging at home would be highly beneficial.

### Lack of Home Care Coordination Tools

Families of CMC could be apprehensive about leaving the clinical setting because of the complexity of their medical care responsibilities. Care coordination services could be supportive and helpful for transitioning after discharge.

...when we were going home (after NICU discharge), I wasn’t ready to go home.

Caregiver

For care coordination services in the home setting, generic emailing and messaging apps and special-purpose nonmedical care apps (reminders for medications, diaper changes, feedings, etc) have been commonly used. However, care coordination apps are limited or nonexistent for complex services, lacking functionalities such as allowing multiple users to communicate and coordinate or providing on-demand coaching of home care skills [14]. Lack of care coordination among caregivers can result in high indirect health care costs and poor outcomes, such as overmedicating or undermedicating, medication errors, safety issues, and emotional stress [15]. Providing relevant and timely instructions on caregiving procedures at home during time of need will also reduce the demand to connect with care coordinators, build home care skills, and avoid costly mistakes. A patient-centered and easily accessible tool that facilitates coordination among home caregivers would reduce miscommunications, delays, and stress, thus reducing costs resulting from errors and improving outcomes.

It is common practice to use a verbal or informal note as a *handoff* to communicate during transitions between caregivers. However, this requires extra effort and coordination, as there can be adverse events when the notes are not written, illegible, lost, or not noticed. Transcribing handwritten notes manually into patient records is also cumbersome, time consuming, and error prone. This inefficient flow of information during transition times may lead to additional caregiver burden and reduced quality of care [16]. The use of digital tools, such as mobile phone apps and Web services, would be preferable but might be inconvenient if the interaction with the apps requires the physical and visual focus to shift away from caregiving activities. Most of the current digital tools lack integration with EMR systems, which prevents timely 2-way communication between provider and caregiver. Critical health information captured at home should flow seamlessly into EMR for a timely response from the clinical team. EMR systems are preferred as the major hub for personal health information. However, they are designed primarily to capture encounters with providers and to bill for services. Most EMR systems do not provide useful tools for a patient-initiated medical diary. Most patient-facing EMR utilities provide access to limited clinical information, sending or receiving an email to/from one provider at each instance, and completing predesigned health assessment questionnaires. Often, parents of CMC need to discuss a problem with multiple providers, who also need to discuss among themselves. For instance, if a child receiving in-home ventilation experiences respiratory symptoms, the parent may want to inform and discuss these symptoms with the primary care provider, the pulmonologist, and the otolaryngology surgeon at the same time and on the same thread, which would improve the efficiency of resolving the problem.

Unfortunately, there is limited literature addressing care coordination technologies and their utilities for CSHCN or CMC and caregivers in home setting. A majority of the studies focus on the use of health communication technologies (Web-based and mobile tools) in self-care, and the results show limited evidence regarding care coordination outcomes [17].

### Long-Term Adoption Problem for Health Care Apps

The most commonly used and accessible health care technologies are mobile apps. In general, long-term sustained usage of health care apps is low [4].

The app takes too many taps to get to the right screen. I stopped using it after a month...

Caregiver

Many existing apps have the potential to deliver great value to end users but have failed to keep users engaged long enough to reach that potential [18]. This long-term adoption problem is notable in user ratings and comments of apps that are currently available. Many hours go into the development, marketing, and maintenance of these apps. If the apps are not used long term, there is a great deal of waste involved. Keeping the patient and families engaged for the long term is critical to maximize the true adoption and value of a health care tool. Considering real-world scenarios, improving the convenience of utilizing the technology should increase adoption and sustained engagement in the home setting.

On the provider side, EMR systems have become a common tool with digitized clinical records, and their use has been mandated. However, patient engagement and caregiver engagement depend on the perceived value and whether value outweighs burden of use. Today, many caregivers use paper or other analog, nonunified, unshared, nonsystematic methods to capture medical events and subsequently rely on one-to-one direct communication with providers to coordinate care. An alternate strategy to promote communication would be to make it easier for caregivers to report and capture medical events. Millions of homes have adopted voice interactive devices, such as Amazon Alexa, because they are easy and convenient to use. Voice-enabled technology can be leveraged to more accurately report medication compliance, event documentation, and care coaching. In line with that, our previous study demonstrated that voice interactive technologies are expected to promote adherence in health tracking and increase adoption of communication technologies for care management among caregivers and CSHCN [19]. Toward the effort to reduce the adoption problem, we offer an alternative method to mostly manual and error-prone methods in home care, such as delayed event note taking. We hypothesize that gradual replacement of current in-home methods with the use of tailored and low-burden technologies, such as audio-interactive ambient communication tools, in the home setting could potentially increase effectiveness of care management and coordination for CMC.

## Recommendations

To address the previously identified gaps in the current apps and tools landscape, it is essential to engage all stakeholders of CMC using human-centered design principles to create an accessible and interoperable solution. Our multistakeholder team did not focus on finding the *silver bullet*, but rather, it focused on identifying a potential solution to nudge patients, caregivers, and medical providers in a direction that will achieve better coordination at home. Focusing on reducing challenges, blending in with daily routines, increasing engagement, enabling convenient communication, and tracking/coaching in the home setting, the team recommends the adoption of voice interaction technologies for in-home documentation and information delivery to enhance ease of use and technology adoption.

### Leverage Voice Recognition and Interaction

Voice interactive devices and apps are currently embraced and used in daily life by millions of people. The technology is not a passing eccentricity but rather has multiple embodiments from major technology companies, including Amazon Alexa, Google Assistant, Apple’s Siri, Samsung’s Bixby, and other Internet of Things– and mobile-based platforms. These apps have just started gaining attraction in health care [20]. There are studies investigating the feasibility of voice-activated devices and voice assistants in medical data collection and accuracy in understanding medical terms [21,22]. In addition, research has shown that users are increasingly adopting voice interactive devices and apps that can blend seamlessly into their daily lives [23]. Considering the ease of using in voice interaction to access Web-based information, the adoption of these new technologies may be higher by caregivers [24]. However, documented use of voice interaction in care coordination is limited, especially in pediatric care. Multimedia Appendix 1 summarizes some of the voice interactive tools currently available in the market for care assistance.

Voice interaction is particularly successful when the nature of interaction does not require any visual or tactile feedback, thereby removing personal attention to the device. Users simply speak to the app naturally, and information will be captured or recorded. The app can allow caregivers to provide details of symptoms and health events in the most natural and narrative way, enabling hands-free voice interactivity, which might be critical for people who have physical limitations and are not able to type in information. Shifting to an audio diary with voice interaction could increase adherence in keeping a log, specifically when a diary is prescribed to record the frequency of seizures, follow-up with diabetic laboratory tests, or to track general medical symptoms.

The ease of leveraging natural voice documentation needs to be supported with strong natural language processing (NLP) for both voice transcription to text and information extraction from the unstructured text. NLP, together with advanced data science methodologies, has been developed and continues to be improved to take full advantage of the richness of contextual information presented in natural narratives [25]. It can be used to extract relevant information related to symptoms associated with diagnoses, identify signs of worsening conditions, and record medication compliance, all to facilitate the care coordination process. Given the current practices utilizing machine learning in digital health, user differentiation and identification, pattern and characteristic recognition, medical alert and prescription reminders, and emerging needs prediction are all potential scenarios once an adequate amount of voice data are acquired for training the algorithms. Collectively, speech and audio inputs have the potential to be used as digital biomarkers in the future for detecting and predicting disorders, diseases, and acute deterioration events [26].

### Extend the System Architecture to Incorporate Voice Interactive Technology

**Figure 1:**
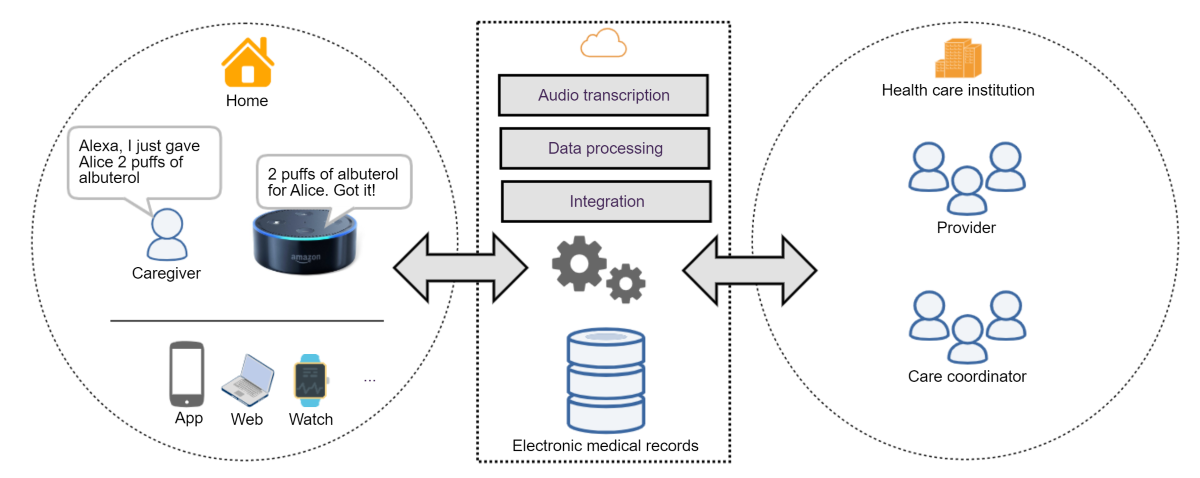
An ecosystem of voice interactive care coordination unifying homecare with health care institutions. EMR: electronic medical record; IoT: Internet of Things.

The recommended solution framework is illustrated in Figure 1. In the home, a patient and caregiver can interact with the solution system via voice, allowing convenient engagement in a naturalistic setting. The voice interaction would facilitate information exchanges among devices or components in the user network, promoting a *personalized digital ecosystem* (services such as If This Then That and Apple Health enable such information exchange). The data will be processed (eg, voice can be transcribed and converted into text with established and secure services such as Amazon Transcribe or SiriKit; text can be further processed into structured data using NLP services such as Amazon Comprehend); information collected can be integrated with clinical care and management systems at the backend using interoperable standard of fast health care interoperability resources. The proposed system can be implemented to make summarized and curated data available to providers and care coordinators at the next visit. Considering real-world use cases and current needs, we have anticipated key functionalities that would be required. Features have been grouped according to 3 identified challenges (Textbox 1). Textbox 2 provides 3 real-world user stories suggesting the potential use of voice interaction in care coordination.

**Example functionalities of voice interactive tool.**

1. Timely capture of complete and accurate health information at home

- Voice enabled for natural unstructured, real-time health information capture
- Can record audio (such as coughing) and video to communicate with care provider team
- User validates and edits text transcribed from voice to address transcription inaccuracy and privacy
- Direct free-text documentation is also available to accommodate multiple input modalities
- Register care needs using trigger words to notify the care coordination team

2. Facilitate coordination among caregivers at home

- Allow multiple users with different level of access
- Segment health information to reflect privacy preferences (public, shared, private, etc)
- Voice-enabled retrieval of recent care history using predefined trigger keywords
- Provide instructions or coaching on relevant home treatment procedures

3. Foster adoption of the app and long-term engagement of users

- Integrate with electronic health record to pull clinical information and push home care information back
- Enroll patients to use this app and help them to set up linkage at clinical visit
- Reminders for medications, next scheduled visits, and updating of symptoms
- Customized reports to patients periodically to provide value to them and to keep them engaged
- Leverage Health Insurance Portability and Accountability Act–compliant Web servers and services for data storing and analysis
- Raw captured data are distilled to represent succinct and relevant historical clinical information

4. Integrate the solution with the health care delivery system for care coordination

- Receive feedback from the care coordination center
- Adopt fast health care interoperability resources application program interfaces for interoperability
- Demonstrate integration with a health care delivery system including care coordination

**User stories with voice interaction in care coordination.**

**User story 1: Incomplete information on symptoms and health events at home**

- Who: Parent of a child who has asthma
- What: Cannot recall how often he has been given Albuterol in the last 3 months and how many times the child has woken up because of night time coughing
- Why: The above missing information is needed to assess asthma severity and recommend the right treatment plan
- Solution:
  - Using the app, the parent documented the child’s asthma symptoms and treatment as they occurred.
  - The parent clicked the links to review and update Albuterol dosage and treatment times. Also, increased coughing events at night were noted.
  - A week later at the doctor’s appointment, the parent filled out questionnaire on symptoms referencing the records in the app.
  - Doctor: “Do you have any concerns over the last 3 months?”
  - The parent pulled out the app to review the list of concerns and the relevant symptom histories

**User story 2: Complexity of care coordination among caregivers**

- Who: Parents and a child with multiple health problems including cerebral palsy, epilepsy, tracheostomy, and gastrostomy who uses a wheelchair
- What: Have trouble coordinating complex care at home (tracheostomy tube changes and medication administration, etc)
- Why: Not knowing whether the previous caregiver has given antiepileptic, at what time and dosage, if it could be dangerous and negatively impact health outcome
- Solution:
  - Mom: “Alexa, Depakote 5mL given to Ben” (timestamp captured and recorded)
  - Amazon Alexa: “Depakote 5mL given to Ben. Got it”
  - Mom “Alexa. Ben Trach changed”
  - Amazon Alexa: “Trach changed for Ben. Got it”
  - Dad: “Alexa. When was the Trach changed last for Ben?”
  - Amazon Alexa: “Trach was last changed at 3:05 pm today for Ben.”

**User story 3: Stress of care coordination between youth and parents**

- Who: A diabetic teenager who needs daily insulin shots and caring parents
- What: The teenager gets agitated when parents check in daily to make sure medications are taken
- Why: The teenager perceives parents’ medication monitoring and reinforcements as nagging
- Solution:
  - Teenager: “Hey Siri. Insulin given” (timestamp captured and recorded)
  - Apple watch: “Got it. Insulin given at <current time>”.
  - Parents are also authorized to see the records and would need to “nag” the teenager a lot less, resulting in less stress and better teenager-parent relationship.
  - The teenager can also view her compliance of treatments and glucose levels over time. She begins to take responsibility for monitoring her own health but continues to have oversight by parents.

### Define and Measure Outcome Metrics

Outcome metrics for voice interaction could be slightly different from other technologies because of the nature of information processing and technology interaction. Therefore, it is important to consider the differences and adjust the metrics. In voice interaction, outcome metrics could be collected and assessed in 2 categories: technical and engagement. To validate the technical performance of the technology, accuracy testing of artificial intelligence (AI) and NLP methods would be employed. Annotated notes, number of user-validated transcriptions and notes, and number of retaken or corrected notes could be used to test the performance of AI. Precision and recall rates could be used to assess the accuracy of NLP methods in predicting and providing note highlights. *Engagement* assessment could be done for adoption of technology services and utilization of knowledge provided. The quantitative log data, such as number of users per patient (eg, care coordinator, medical provider, or caregiver), audio notes taken, number of transcriptions reviewed or edited, and number of parent instructions used, could be employed to assess user adoption. In addition to that, usability testing of voice interaction to understand the narratives, co-design sessions for creating the voice interface, and technology adoption interviews and surveys could be utilized to comprehensively analyze the adoption and correlating factors. Utilization of the knowledge provided would be assessed in the long term, such as through comparison of families using digital health versus nonusers in terms of emergency department visits and hospitalizations. The *observable* results based on the use of suggestions at home would imply utilization by patients and caregivers. The number of times speech-to-text notes are pulled from the EMR and reviewed by providers would be indicators of utilization by the providers. The assessment of the outcome requires multilayered and multitheoretical research plans to understand the impact and implications of the proposed technology.

### Potential Limitations and Implementation Challenges

**Figure 2:**
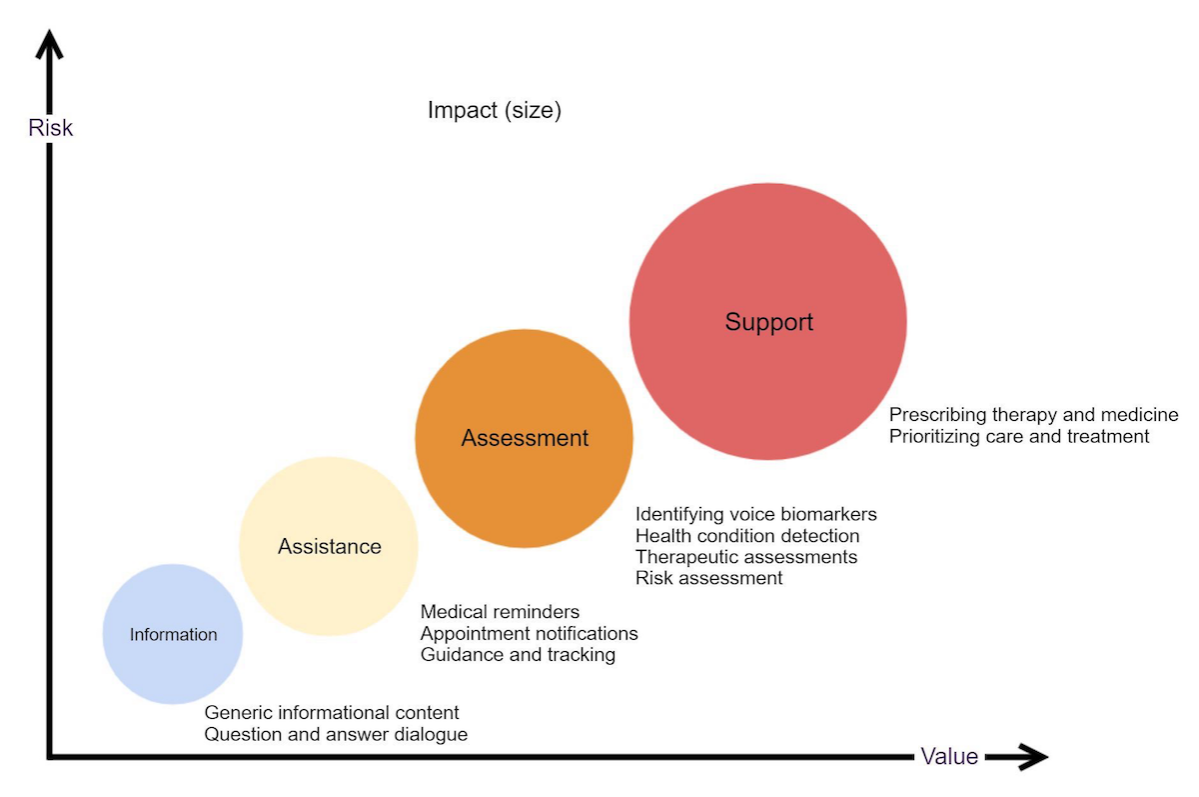
Spectrum of applications of voice assistants.

As shown in Figure 2 (Adapted from [27]), the utilization of voice interactive services is in the early stage, and it would eventually advance from information-level services (eg, educational content and internet search) to assistance (eg, guidance and instruction, reminders, and alerts and tracking), then to assessment (eg, identification and detection, prediction with biomarkers, and management), and eventually to support (substituting or supplementing the medication and therapy tools). Currently, the implementations are moving from *information* to *assistance* level in a low-risk and limited-service approach, such as medical reminders and other messages used only in the hospital setting, similar to the current state of voice assistants in self-management [28]. However, as the use of voice interactive devices and services grows, both their impact on health care and the risks on privacy and security increase.

Within this context, voice assistants in care coordination, as the envisioned solution in this paper, may have implemental, ethical, regulatory, and technical limitations. Although significant progress has been made in terms of compliance of services (eg, Health Insurance Portability and Accountability Act [HIPAA] compliance of Amazon Alexa), consumer-facing voice interactive device apps currently have limited abilities to be used in health care. Some of the emerging limitations for leveraging voice are as follows:

- Mainstream vendors are not providing full access and control of the voice input (eg, transcript and raw data) to developers and researchers with user consent, which can be used for improving health services.
- Not all health care services are HIPAA compliant and have limited security and privacy protocols related to audio-formatted health data transmission, processing, and storage.
- There is relatively lower demand in the market compared with other communication technologies (eg, mobile apps).
- Access to voice-enabled devices is affected by the social economic status and may create inequality in access to the solution (eg, requirement for compatible device and data plan).
- There is a major progress in voice recognition in the English language but limited efforts on foreign-accent recognition and lack of availability and analytical capability in a large selection of other languages.
- New methods are needed for designing voice services in health care. Translating mobile or Web services to voice may be limited in terms of functionality and navigation.

Conversely, integration of unstructured patient-reported data with the health care system could create a systematic burden and may be hard to control and use in decision making [29]. These unstructured data need to be coupled with NLP and AI to extract and present the relevant information to the providers. This is not a new problem as physician’s patient notes constitute the majority of unstructured data within the EMR [30]. However, integrating care and health information collected at home needs strategic planning and providers’ buy-in. At a time when physician burnout and alert fatigue are such pressing issues, additional information streaming from a patient’s home into the EMR needs to be distilled to support clinical decision making [31]. In addition, clinical workflow may need to be modified to balance the tradeoff of a timely response to urgent issues and to reduce potential clinician burnout.

Considering the increasing investments in health care and voice technologies and the current trajectory of voice interactive device adoption [23], it is expected that voice interactive platforms will have an impact as household health communication tools or as telemedicine tools in the long term. Limitations could be mitigated by policy changes such as reducing cost and increasing accessibility by potentially collaborating with accountable care organizations [32]; promoting employer-based health insurance coverage for voice assistants; and inclusion in digital health policy and regulations, such as the Food and Drug Administration’s Digital Health Innovation Action Plan [33].

## Conclusions

In this paper, we have shared the challenges and recommendations regarding the use of technology to promote coordination of the care of CMC in a home setting. We argue that the use of voice interactive technologies in the home setting could enhance communication of health events and improve coordination. Although the current literature is limited in relation to voice assistant use in care, our report contributes to the literature suggesting potential health informatics solutions, which address information needs for coordination [7].

## Appendix

Multimedia Appendix 1 Voice interactive assistants in the market.

## Abbreviations

AI: artificial intelligence
CSHCN: children with special health care needs
CMC: children with medical complexity
EMR: electronic medical record
HIPAA: Health Insurance Portability and Accountability Act
MCHB: Maternal and Child Health Bureau
NLP: natural language processing
