# Supplementary material for: Capturing At-Home Health and Care Information for Children With Medical Complexity Using Voice Interactive Technologies: Multi-Stakeholder Viewpoint
Source: J Med Internet Res. 2020 Feb 13;22(2):e14202. doi: 10.2196/14202 (PMC7055855; doi:10.2196/14202)
Supplement: Multimedia Appendix 1 [file jmir_v22i2e14202_app1.docx]

Multimedia Appendix 1 - Voice interactive assistants in the market

| **Product** | **Description** | **Accessibility/ Availability** | **Voice interactive?** | **Free text entry allowed?** | **Features** | **Platform** |
| --- | --- | --- | --- | --- | --- | --- |
| Updoc: [Health Diary](https://play.google.com/store/apps/details?id=com.updochealth.updochealth) | Personal medical diary app. It is text-based, do not use voice input and transcription | Available on Android and iOS | No | Yes | - Track symptoms - measurements, medication and examinations - Add photos of symptoms or exam results Check progress through graphs Export and share logs | Mobile apps |
| [Daybook](https://play.google.com/store/apps/details?id=com.bigheadtechies.diary) | Personal diary designed to record activities, experiences, thoughts and ideas throughout a day. Not focused on healthcare. | Available on Android and iOS | No (only entering notes) | Yes | - Speech to text entry - Image/video entry - Scheduling events |  |
| [VICA](https://www.krminc.com/portfolio/voice-initiated-care-assistant-vica/) (Voice Initiated Care Assistant) | A voice assistant for accessing drug side effects and information, reviewing medication schedules, capturing clinical data / survey, potential skill to read discharge instructions, using a care management platform to retrieve/store data | Proof-of-concept | Yes | No | - Drug side effects and information review - Medication schedules - Capture clinical data and survey | Voice-interactive device applications |
| [Northwell Health skill](https://www.northwell.edu/about/news/press-releases/ask-alexa-wait-times-locations-northwell-ers-urgent-care-centers) | It is an emergency and urgent care wait time portal, which analyzes check-ins at Northwell EDs and updates them every 15 minutes. | Limited access on Amazon Alexa | Yes | No | - Emergency location sharing - Wait time updates |  |
| [Libertana Home Health](https://www.amazon.com/Libertana-Home-Health/dp/B07C29YFGD) | A voice assistant for elder residents to develop self-management skill. For now, the primary functions are to give residents a rundown of their daily schedule, remind them to take medications and required health checks – and to log that information for caregivers | Available in Amazon store | Yes | No | - Tracking daily activities - Reminders of tasks and/or calendar events - Send/Receive messages with caregivers |  |
| [CardioCube](https://cardiocube.com/) | Home voice assistant that patients can request doctor appointments, medication refills, visitation check-ins and send updates about their conditions that facilitates disease management and communication | Limited access on Amazon Alexa | Yes | No | - Scheduling appointment or rescheduling visit - Sharing patient-reported medical information - Daily conversations for disease management. - Medication refill information - Reminders - Requesting referrals - Q&A with care team |  |
| [MyChildren's Enhanced Recovery After Surgery](https://www.amazon.com/MyChildrens-Enhanced-Recovery-After-Surgery/dp/B07QB7PQYW) | Skill supports parents and caregivers after discharge who have children undergone heart surgery. It helps to provide updates to the provider about recovery progress. Skill also provide information to caregivers about scheduled post-op appointments. | Available in Amazon Store | Yes | No | - Sending quick updates to care team - Receiving information for scheduled post-op appointments |  |
